# Attitudes to and perceptions of workplace health promotion amongst employees from ethnic minorities in the UK: A scoping review

**DOI:** 10.3233/WOR-230576

**Published:** 2024-09-11

**Authors:** Katharine Platts, Emma Scott, Kerry Griffiths, Anouska Carter

**Affiliations:** a Sheffield Hallam University, Sheffield, UK; b University of Warwick, Coventry, UK

**Keywords:** Workplace, health promotion, ethnic and racial minorities, United Kingdom, occupational groups, qualitative research

## Abstract

**BACKGROUND:**

Ethnic minorities make up approximately 14% of the UK workforce. Despite the disproportionate burden of ill-health amongst ethnic minorities, and the increased interest in Diversity, Equity & Inclusion (DE&I) in the workplace, workplace health and wellbeing interventions are still most often designed for the ethnic majority.

**OBJECTIVE:**

The purpose of this scoping review was to explore the depth and breadth of evidence on the attitudes to and perceptions of health and wellbeing interventions in the workplace within ethnic minority groups in the UK, and to identify gaps in evidence that would provide direction for future research needs.

**METHODS:**

A scoping review with quality appraisal was undertaken, supplemented by a review of grey literature and a narrative review exploring related evidence from the knowledge bases related to community and cultural adaptation.

**RESULTS:**

Only three peer-reviewed studies met inclusion criteria, preventing broad conclusions. 14 papers from the community and cultural adaptation literature provided additional information about how health promotion may be approached effectively in the workplace, including the importance of culturally sensitive, people-centred design, and the use of established adaptation frameworks.

**CONCLUSION:**

The literature suggests a need for improvements in four key areas: (1) reporting of ethnic minorities in data relating to workplace health and wellbeing research, (2) more thorough review of perceptions and attitudes of ethnic minority workers in the UK, (3) design of culturally appropriate interventions that are tested for impact, and (4) testing of the effectiveness of culturally adapted interventions.

## 1 Introduction

Research and services are predominantly designed to support the population majority. Yet ethnic minorities make up 18.3% of the UK population (approximately 10.9 million people), with South Asian and Black populations making up the largest ethnic groups in England and Wales [1].

Ethnic minorities in the UK are reported to experience a disproportionate burden of health inequalities [2], with increased risk of diabetes [3, 4], cardiovascular disease [5, 6] and mental health issues [7]. Stigma towards mental illness is also reported to be higher among ethnic minority groups [8].

Ethnic minorities make up approximately 14% of the UK workforce (approximately 4 million people [9]). Dame Carol Black’s landmark review in 2008 identified the workplace as key setting for health and wellbeing improvement [10]. More recently, the issues of an aging workforce, and increased exit of people over 50 years old with long-term conditions from work, have thrown the need for comprehensive workplace wellbeing support into sharp focus [11]. Although these reports do not focus on ethnic minority groups specifically, the workplace offers a potential environment in which to influence and improve overall health and wellbeing in minority groups across the UK. It is therefore imperative that the provision of health and wellbeing services in the workplace provide equal support to all sections of the workforce, including those from ethnic minorities [12].

There has been an ongoing interest in enhancing wellbeing in the workplace, boosted significantly by the Covid-19 pandemic which has forced employers to consider different working practices and environments for their employees [13]. Despite the disproportionate burden of ill-health amongst ethnic minorities, and the interest in Diversity, Equity & Inclusion (DE&I) in the workplace, workplace health and wellbeing interventions are most often designed for the ethnic majority [12].

The focus on better supporting the health and wellbeing of ethnic minorities in the UK workforce is an emerging field, with limited literature. Exploration of this issue via a scoping review provided a flexible approach that would allow inclusion of grey literature and adaptation of the inclusion and exclusion criteria to ensure that all relevant documents could be included.

The main purpose of this scoping review was to explore the depth and breadth of evidence about attitudes to, and perceptions of, health and wellbeing interventions in the workplace within ethnic minority groups in the UK, and to identify gaps in evidence that would provide direction for future research needs.

## 2 Methods

This review followed the six-stage framework for scoping reviews (Table 1) as described by Arksey and O’Malley [14] and Levac et al. [15], with additional quality appraisal of studies included in the final review using the CASP criteria for qualitative research [16].

**Table 1:** Six Stages for conducting a scoping review [15]

Six Stages for conducting a scoping review
1. Identifying the research question
2. Identifying relevant studies
3. Selecting studies
4. Charting the data
5. Collating, summarizing, and reporting the results
6. Consulting knowledge users

A full search of the grey literature was conducted, with an additional search of peer-reviewed literature for supplementary data from community health and wellbeing interventions and cultural adaptation literature, to determine if learnings from other settings may be transferable to the workplace environment.

### 2.1 Scoping review –stages

#### 2.1.1 Identifying the research question

Scoping reviews enable a much broader view of the evidence base, yet Levac et al. [15] recommended the importance of combining this with a more detailed scope to focus the search strategy. The PICO framework introduced by Richardson et al. [17] supports this process when seeking both qualitative and quantitative literature. The research team determined the scope and formulated the research question, which was to ascertain what the evidence could reveal about the experience health & wellbeing of ethnic minorities at work, based on this framework (Table 2).

**Table 2:** PICO framework to determine scope for review and search criteria

PICO	Scope	Search Criteria
Population	UK ethnic minorities in part-time or full-time employment, focusing on larger UK minority groups (using ONS census data)	Multicultural OR Ethnic* OR Minorit* AND
Interest/Intervention	Health promotion or wellbeing interventions	Health Promotion OR Well-being OR Wellbeing OR Wellness AND
Context/Comparator	Studies conducted in UK workplace or relevant to the UK workplace	Employ* OR Workforce OR Workplace OR Corporate AND
Outcome	Studies reporting findings on experiences (attitudes, perceptions, barriers, facilitators) of workplace wellbeing intervention/promotion from the perspectives of ethnic minorities	Attitude OR Perception OR Barrier OR Facilitator OR Experience
Study Design	Longitudinal, experimental, qualitative, pilot/feasibility, mixed methods

#### 2.1.2 Identifying relevant peer review studies and grey literature

Criteria for a full systematic search of relevant citations was decided upon by the research team. Anticipating a low number of relevant papers, the research team opted to include studies that either took place in a UK workplace or that had relevance to the UK workplace. Systematic searches were conducted using PubMed, SCOPUS, and The Cochrane Library. The search criteria are outlined in Table 2.

In addition, three journals highlighted as specifically relevant by the research team were hand searched via contents, title and abstract for further relevant studies. A further two journals were included in the hand search in response to the frequency with which articles from these journals appeared in the reference lists of other review articles on similar topics.

Furthermore, the research team developed a comprehensive list of websites from predominantly UK-based organisations that warranted an independent search for relevant grey literature (Table 3). These websites were searched for literature related to ethnic minorities in the workplace.

**Table 3:** Websites searched for relevant grey literature and findings of relevance

Organisation/website	Findings	Count and relevance
Academy of Royal Medical Colleges (AMRC) https://www.aomrc.org.uk/ / Royal College of Nursing (RCN) https://www.rcn.org.uk/ / Allied Health Professional Federation (AHPF) http://ahpf.org.uk/	1 Consensus Statement	1NRI
British Occupational Health Research Foundation https://www.bohrf.org.uk/	8 Articles	8NRI
Chartered Institute of Personnel and Development (CIPD) https://www.cipd.org/uk/	0
Department of Health, now Department of Health &Social Care (DoH, DoHSC) https://www.gov.uk/government/organisations/department-of-health-and-social-care	1 Report	1NRI
Department of Work &Pensions (DWP) https://www.gov.uk/government/organisations/department-for-work-pensions	1 Report1 Bulletin	2NRI
Faculty of Occupational Medicine, Business in the Community (BITC) https://www.fom.ac.uk/about-us	2 Toolkits	2NRI
Health &Safety Executive (HSE) https://www.hse.gov.uk/index.htm	5 reports	3NRI2PRI
Mental Health First Aid.org. https://www.mentalhealthfirstaid.org/	1 Blog	1NRI
National Institute for Health and Care Excellence (NICE) https://www.nice.org.uk/	35 Reports	34NRI1PRI
Partnership for European Research in Occupational Safety and Health (PEROSH) www.perosh.eu	4 Conference Papers3 Articles	7NRI
Public Health England (PHE), now Office of Health Improvement &Disparities (OHID, part of DHSC) https://www.gov.uk/government/organisations/office-for-health-improvement-and-disparities	1 Report	1NRI
Wellbeing at Work Conferences –coordinated by PEROSH https://perosh.eu/repository/programme-wellbeing-at-work-2022/	4 Conference Papers3 Articles	7NRI
Wellcome Trust Home | Wellcome	1 Report	1PRI
Total	**71**	**68NRI**
		**4PRI**

NRI = Not Relevant Information, PRI = Potentially Relevant Information, RI = Relevant Information.

#### 2.1.3 Selecting studies

References were imported into Endnote and duplicates removed. Titles and abstracts were screened in Endnote, with relevant texts obtained for full text screening. The inclusion and exclusion criteria for screening are presented in Table 4.

**Table 4:** Inclusion and exclusion criteria for article screening

Inclusion	Exclusion
Study took place in a UK workplace or has relevance to the UK workplace	Studies that do not report population by ethnic group
Study includes at least 1 ethnic minority as outlined by ONS	Studies taking place outside the UK with no application to the UK workplace
Studies written in English	Studies not written in English
Study taken place in last 10 years (expand to 20 if needed)	Studies older than 10 years from data of search
FREE full text only

Full-text screening was conducted by two researchers (ES, KP) working independently with any differences of opinion regarding inclusion discussed by a third reviewer (AC). The PRISMA (Preferred Reporting Items for Systematic Review and Meta-Analyses) flow chart was used to report results.

#### 2.1.4 Charting the data

A data charting form was created by the researchers undertaking full text screening (ES, KP) to enable data extraction, with any differences of opinion related to included content resolved by a third reviewer (AC). Key information extracted during this phase is outlined in Table 5. A quality appraisal of the included studies was conducted by a fourth reviewer (KG) using the CASP questions for qualitative literature [16].

**Table 5:** Summary of scoping review citations

Citation/ country of origin	Research l	Design/ methodology	N=	Age	Gender	Ethnicity	Context	Type of intervention	Key findings
Bertotti et al. [18] UK/London	(1) Understand the context and approach to staff well-being within Chinese owned businesses based in London (2) identify any potential levers, barriers, and triggers for engaging Chinese-led businesses with workplace well-being initiatives	Qualitative - Semi-structured interviews and focus groups Cross-sectional Thematic Content Analysis	Interviews - *n* = 11 employees; *n* = 17 employers; focus groups - *n* = 10 employees	Interviews (employees): 25–35 *n* = 7 35–45 *n* = 1 65–75 *n* = 2 Interviews (employers): 25–35 *n* = 1 30–40 *n* = 3 35–45 *n* = 3 40–50 *n* = 4 45–55 *n* = 3 50–60 *n* = 1 55–65 *n* = 2 Focus group: not reported	Interviews (employees): 7 male; 4 female Interviews (employers): 12 male; 5 female Focus group: not reported	Chinese living in London	Chinese SMEs in London	No intervention - cross-sectional exploration of attitudes towards workplace wellness and willingness to engage	•Employers’ attitudes towards workplace wellbeing were reactive rather than proactive, informal, and characterised by in-house on the job health and safety training. But they would make changes if a convincing business case could be made.
									•Few employers demonstrated awareness of the impact of issues such as salary levels, working conditions, workers’ rights, and relationships between colleagues which, in contrast, were key concerns of the employees.
									•Generation of owner - first generation Chinese vs British-born Chinese effects willingness to embrace more western approach to business, including workplace wellness.
Verburgh et al. [19] Netherlands/Amsterdam	What is the impact of the Work-Life Program on women’s health and work functioning? aims to support female workers during menopause and midlife in making choices that will enhance their health and wellbeing in both their working and private lives.	Mixed methods - before and after questionnaire; semi-structured in-depth interviews Longitudinal	Quantitative *n* = 56; Qualitative *n* = 12	Only 45–60yrs old eligible; mean age 52.6 + /–4.5yrs	All Female	Quantitative: Ethnic majority –(Dutch) *n* = 34 Ethnic minority *n* = 36 (21 different backgrounds) Qualitative: Ethnic majority (Dutch) *n* = 5 Ethnic minority *n* = 7	Low paid jobs at Amsterdam University Medical Centre	Integral approach which encompasses an intake session to explore participant needs and general health check, health education on menopause, lifestyle coaching to improve work-life balance, and physical training. 8x 1hr sessions, flexible scheduling over 2–4mths	•Quantitative findings - only menopausal symptoms showed any significant difference between pre- and post-intervention; psychological, somatic, and vasomotor symptoms, depression and overall score all improved. Anxiety and sexual dysfunction did not. No change in work functioning, quality of life or work ability.
									•Qualitative findings - The WLP initiated a process of mental empowerment (defined as a form of self-efficacy) in most participants; participants said they felt stronger and freer. This has been associated with changes in behaviour, physical health, mental well-being and in the workplace.
									•Findings suggest that female workers in low paid jobs experience positive impact from the WLP. It empowers them to make choices that benefit their health and wellbeing both at work and in their private lives. Additional qualitative methods are indispensable for evaluating the impact of an intervention among a very heterogeneous study population.Citation /Country of Origin
Verburgh et al. [20]	How can we reach and engage an ethnically diverse group of midlife women with a low socioeconomic position (SEP) in the implementation of this workplace health promotion (WHP) intervention?	Qualitative evaluation of the implementation of the WLP using the RE-AIM framework (Reach, Effect, Adoption, Implementation, Maintenance). R: Quant plus interviews; E: mixed methods [19]; A: Focus group and interviews; I: interviews; M: focus groupsLongitudinal	Interviews - *n* = 12 Intervention participants; *n* = 5 professionals involved in implementing intervention (out of 10 involved); Focus group - *n* = 6 organisation stakeholders	Only 45–60yrs old eligible; mean age 52.6 + /–4.5yrs	All Female	Ethnic majority (Dutch) *n* = 34 Ethnic minority *n* = 36 (21 different backgrounds)	As Above	As Above	•Reach - Personal invitation letter most influential to participate; information meetings also perceived to have added value, even if they had already decided to participate, especially for those who could not read or fully understand the letter. The presence of line managers of the same ethnic background at verbal invitation meetings was important to create trust.
									•Implementation - Facilitators: (1) accessibility of offering sessions in the workplace and in work time; (2) program was tailor-made and both individual and group sessions were an option; (3) practical support for low literacy and language barriers; (4) female facilitators/professionals especially for women from non-western backgrounds.
									•Implementation - Barriers: (1) practicality of creating time in the workday to attend sessions; (2) inconsistent time interval between sessions; (3) availability/location of rooms for sessions.

#### 2.1.5 Collating, summarising and reporting the results

Following the conventions described by Levac et al. [15], a descriptive summary of the data was prepared, with careful reference to the original research questions and purpose of review, and implications for future research, practice, and policy were considered.

#### 2.1.6 Consulting knowledge users

The research team consulted with stakeholders at a UK-based private sector provider of workplace wellness solutions during the review process to ensure that the reported results and method of reporting had commercial relevance and were suitable and appropriate for organisational use.

### 2.2 Supplementary narrative review

Based on initial exploratory work the authors were aware that the scoping review may only yield a small number of papers. Therefore, to support the workplace context-specific data, a supplementary search was conducted (AC) to enable a narrative review of community and cultural adaptation literature related to health and wellbeing interventions in the UK.

It is acknowledged that a narrative review lacks the scientific rigor of a systematic or scoping review and is subject to author bias. However, it was deemed a useful method for obtaining additional information of relevance and gaining a wider perspective on the research topic.

For the supplementary narrative review the team looked at literature relating to cultural adaptations of health and wellbeing interventions in the community that may have relevance to the workplace. The following databases were searched for literature relevant to answering the research question, PubMed, SCOPUS, and Google Scholar. A combination of the following keywords was selected to be used: ‘community’, ‘ethnic’, ‘minority’, ‘wellbeing’, and ‘health’. The additional terms were added following an initial review of findings: ‘lifestyle’, ‘physical activity’, ‘nutrition’, ‘cultural adaptation’. Data was not restricted by publication date but was restricted to papers published in English and readily available to review.

## 3 Results

### 3.1 Scoping review

#### 3.1.1 Identifying and selecting articles

##### 3.1.1.1 Academic literature

**Fig. 1:**
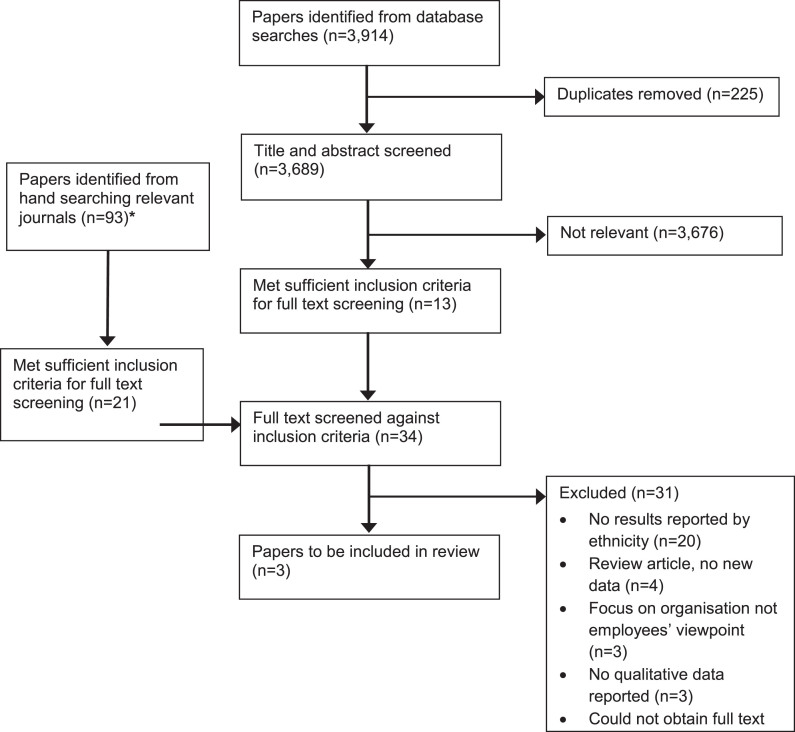
Adapted PRISMA flow chart. *Journals hand searched: International Journal of Workplace Health Management (9 papers found), Journal of Occupational and Environmental Medicine (5 papers), American Journal of Health Promotion (5 papers), Ethnicity and Health (56 papers), Journal of Racial and Ethnic Health Disparities (18 papers).

Initial database searches yielded 3,914 results, after duplicates were removed 3,689 were retained for title and abstract screening. Following title and abstract screening, 3,676 articles were deemed not relevant, with 13 retained for full-text screening. A further 21 articles were retained for full text screening from hand searched journals, leaving a total of 34 papers for full-text review. Only three articles met the criteria and were included in the final review. Results are outlined in Fig. 1 using an adapted PRISMA flow chart.

##### 3.1.1.2 Grey literature

A total of 15 websites were searched for grey literature. Following the website searches 71 pieces of grey literature were reviewed to determine if they contained any relevant information. Types of content included reports, bulletins, consensus statements, conference proceedings, articles, toolkits, and blog posts. Following full screening, only four pieces of grey literature were deemed potentially relevant, none had direct relevance to ethnic minorities experience in the UK workplace. Table 3 outlines the data from each web search.

#### 3.1.2 Charting, collating and summarising results

##### 3.1.2.1 Scoping review programme characteristics

The journal articles were published between 2017 and 2022. Although three articles were included, two were based on the same intervention, looking at different elements of the same study. Only the study by Bertotti et al. [18] was based in the UK and focused on Chinese business in London, whilst the study by Verburgh et al. [19, 20] was based in a Dutch Medical Centre in the Netherlands. Both studies employed qualitative methods using both interviews and focus groups to explore barriers and facilitators to workplace wellbeing. A summary of included citations is provided in Table 5.

##### 3.1.2.2 Quality appraisal

A quality appraisal of the academic literature was carried out using the CASP questions for qualitative data (Table 6) [16]. All papers were of moderate to high quality, with the paper by Bertotti et al. [18] scoring 14/20 and the two Verburgh et al. papers [19, 20] scoring 17 and 18 respectively.

**Table 6:** Key quality appraisal results using the CASP questions [16]

Paper/CASP questions	Bertotti et al. [18]	Verburgh et al. [19]	Verburgh et al. [20]
1. Was there a clear statement of the aims of the research?	Yes	Yes	Yes
2. Is a qualitative methodology appropriate?	Yes	Yes	Yes
3. Was the research design appropriate to address the aims of the research?	Yes	Yes	Yes
4. Was the recruitment strategy appropriate to the aims of the research?	Unsure	No	Unsure
5. Was the data collected in a way that addressed the research issue?	Unsure	Yes	Yes
6. Has the relationship between researcher and participants been adequately considered?	No	Yes	Unsure
7. Have ethical issues been taken into consideration?	Yes	Yes	Yes
8. Was the data analysis sufficiently rigorous?	Unsure	Yes	Yes
9. Is there a clear statement of findings?	Yes	Yes	Yes
10. How valuable is the research?	Unsure	Unsure	Yes
**TOTAL SCORE/20 (Yes = 2, Unsure = 1, No = 0)**	**14**	**17**	**18**

##### 3.1.2.3 Grey literature

The grey literature search revealed four reports/articles that contained potentially relevant information. None were directly relevant, and most of the workplace grey literature had no mention of race or ethnicity. The four reports/articles are summarised in Table 7.

**Table 7:** Summary of grey literature from scoping review

	Title	Possible transferable findings
Health and Safety Executive (HSE) [22]	RR242 –*The evaluation of occupational health advice in Primary Care (2004).*	Focus on ethnic breakdown of access to primary care, such as reasons for consultation, frequency of contact etc. Features data from London and Sheffield sites, London cohort much more ethnically diverse.
National Institute of Clinical Excellence (NICE) [23]	*Mental wellbeing at work (NG212; March 2022)*	Mention of ethnicity in the Recommendations for Research which asks:
		•What specific needs of employees from different groups (such as income levels, ethnic groups, male or female groups, and age groups) need addressing to facilitate access to individual-level interventions?
		•How effective are individual-level interventions across different groups (such as income levels, ethnic groups, male or female groups, and age groups)?
Wellcome Trust [24]	*Putting Science to Work* –*Where next for workplace mental health?* (2022)	Highlights the lack of evidence looking at how workplace wellness interventions may work (or not) for people of different ages, genders, ethnicities, and socio-economic groups. Recommends further work in this area.
Health &Safety Executive (HSE) [42]	*RR221 - Review of the occupational health and safety of Britain*’*s ethnic minorities (2004)*	US studies suggest that ethnic minorities experience greater levels of workplace stress due to discrimination and harassment. Identifies only a few papers related to occupational health promotion programmes, which are all US-based studies. These point to issues including low enrolment and language barriers.

#### 3.1.3 Consulting knowledge users

The research team prepared a draft project report for the private sector collaborator, with a meeting arranged to disseminate, discuss, and interpret key findings via a presentation to key stakeholders from the business, prior to finalisation of the results.

### 3.2 Supplementary narrative review

#### 3.2.1 Cultural adaptation of health interventions in the community

Database searches yielded a total of 10 papers from the community literature and four papers from the cultural adaptation literature with relevance to the perceptions and attitudes of ethnic minorities to health and wellbeing interventions in the community (Table 8).

**Table 8:** Summary of citations supporting supplementary narrative review

Citation	Title	Key findings
Such et al. [25]	A formative review of physical activity interventions for minority ethnic populations in England	Physical activity interventions targeted at BME groups generally focus on inactivity.
Castro et al. [26]	Issues and Challenges in the Design of Culturally Adapted Evidence-Based Interventions	Evidence regarding the effectiveness of cultural adaptations is mixed. Cultural adaptations are more effective in some sub-cultural groups than others.
Bernal et al. [27]	Ecological validity and cultural sensitivity for outcome research: issues for the cultural adaptation and development of psychosocial treatments with Hispanics	Presents a framework consisting of eight dimensions (language, persons, metaphors, content, concepts, goals, methods, and context) which can support development of culturally sensitive interventions and adaptation of existing psychosocial treatments for specific ethnic groups.
Netto et al. [28]	How can health promotion interventions be adapted for minority ethnic communities? Five principles for guiding the development of behavioural interventions	Identifies five principles for adapting behavioural interventions for minority ethnic communities, in brief as follows: (i) use community resources; (ii) address barriers to access and participation; (iii) develop sensitive communication strategies; (iv) work with cultural or religious values that influence behaviour change; and (v) accommodate cultural identification.
Vincze et al. [29]	Cultural adaptation of health interventions including a nutrition component in Indigenous peoples: a systematic scoping review	Evaluated 66 health promotion interventions aimed at indigenous populations that included a nutrition component. Less than half the studies evaluated involved indigenous participants at a deep level. Visual adaptation strategies were the most commonly reported.
Self et al. [30]	Cultural adaptations of motivational interviewing: A systematic review	Culturally adapted Motivational Interviewing performed significantly better at influencing the primary outcome of interest in multiple studies.
Greenhalgh et al. [34]	Health beliefs and folk models of diabetes in British Bangladeshis: a qualitative study	This study found little evidence of a deterministic attitude to prognosis associated with diabetes in Bangladeshis, and that material barriers to behaviour change carried similar weight to cultural barriers.
Grace et al. [35]	Prevention of type 2 diabetes in British Bangladeshis: qualitative study of community, religious, and professional perspectives	Religion has an important part to play in supporting health promotion in the British Bangladeshi community. Collaboration between educators, religious leaders and health professionals may be key.
Choudhury et al. [36]	Understanding and beliefs of diabetes in the UK Bangladeshi population	Bangladeshi participants in this study were found to be quite passive about their own diabetes self-management and relied strongly on the doctor’s views and recommendations.
Gumber [37]	Knowledge gaps and other barriers in type 2 diabetes management: Findings from interviews with South Asian women	Diet, physical activity, and language are the main hurdles that face South Asian women with diabetes. Bilingual services would help to reduce the knowledge gap that some women have related to diabetes development and control.
Alam et al. [38]	A scoping review on the experiences and preferences in accessing diabetes-related healthcare information and services by British Bangladeshis	This review found that language and literacy issues were the most common access barriers to diabetes information and services for British Bangladeshis. Religious and cultural beliefs also play an important role in accessing information and services for this population.
Sohal et al. [39]	Barriers and Facilitators for Type-2 Diabetes Management in South Asians: A Systematic Review	Facilitators identified for diabetes management in South Asians included trust in care providers, use of culturally appropriate exercise and dietary advice, and increased family involvement. Themes for the barriers included lack of knowledge and misperceptions.Citation
Ige-Elegbede et al. [40]	Barriers and facilitators of physical activity among adults and older adults from Black and Minority Ethnic groups in the UK: A systematic review of qualitative studies	This review of 10 studies identified six key themes associated with physical activity in UK BME groups: (1) varying awareness of the links between physical activity and health, (2) levels of engagement with health professionals, (3) cultural / social expectations, (4) a suitable environment, (5) religious fatalism, (6) practical challenges.
Majumdar et al. [41]	Facilitators and barriers to making healthy lifestyle choices: a qualitative exploration in a UK-based Ghanaian population	Three themes associated with healthy eating, healthy weight, and engagement with health care were identified in this study: (1) cultural eating practices, (2) interpretation of national guidelines as “foreign and inapplicable”, and (3) the influence of perceived stereotyping and prejudice.

## 4 Discussion

### 4.1 Main findings

Despite workplace wellbeing receiving a lot of attention in the UK over the last 15 years, and the more recent highlighted importance of DE&I in the workplace, there has been very little academic or grey literature directly reporting on the attitudes and perceptions of workplace wellbeing of ethnic minority workers in the UK. Only three papers met the scoping review inclusion criteria, only one of which was UK based, targeting Chinese employers in London. Whilst the other was European and included a large number (*n* = 21) of different ethnic groups.

Data from grey literature was also sparse, with only four reports/articles containing reference to race or ethnic minorities, none of which had information that directly answered the reviews research question. Further supplementary literature from community interventions, highlighted some useful findings, but again there was a lack of robust trials to test the efficacy of interventions adapted for different ethnic minority groups.

### 4.2 Scoping review

#### 4.2.1 Peer reviewed articles

Findings from the paper by Bertotti et al. [18] suggested that Chinese Employers in London had a reactive approach to health and wellbeing at work and would need a convincing business case to change practices. Views were affected by whether the business owners were first generation Chinese or British born, with the latter more willing to take on Western business approaches including workplace wellbeing policies and resources. Employees in this study were all Chinese, and problems highlighted included poor mental health and poor working conditions and wages –something the employers seemed unaware of.

This paper identified fundamental issues that needed to be addressed in Chinese-owned businesses that included basic health and safety, as well as a lack of workplace wellness engagement. Participants were from a limited business sector of English-speaking businesses, who volunteered to take part, so these results may not be generalisable to Chinese workers in other non-Chinese owned businesses. Indeed, other Chinese owned businesses who did not volunteer may have business environments that are more or less supportive of worker health.

Only the paper by Verburgh et al. [19] was based around an intervention ‘the Work-Life Program (WLP)’, which explored the impact of the programme on women’s health and work functioning in a Dutch medical centre. The programme demonstrated impact, with menopausal symptoms significantly improving following the intervention despite work-related parameters remaining unchanged. The qualitative findings suggest that female workers in low paid jobs experienced a positive impact from the WLP. The WLP was reported to empower them to make choices that benefit their health and wellbeing both at work and in their private lives, through a process of mental empowerment.

Although there is limited insight regarding reasons for engagement in the trial, participants highlighted that the WLP offered opportunities not usually available to them in their culture, specifically the discussion of menopause, midlife changes, and their own needs. Participants were not recruited based on any specific issues related to the topic of interest and came from diverse ethnic backgrounds preventing results from being representative across a particular population.

The second paper by Verburgh et al. [20] reported results relating to reaching and engaging women from ethnic minorities in workplace health promotion, although the minorities under study were categorised as ‘mixed’ and were not specified by ethnicity. The study design included many cultural adaptations, which may have aided recruitment and engagement. Recruitment activities, including personalised letters and an additional information meeting with a line manager of similar ethnic background present. This led to a diverse range of women from different ethnic backgrounds participating in the programme.

The key programme facilitators were reported to be: (1) accessibility of offering sessions in the workplace and in work time; (2) programmes tailor-made and both individual and group sessions available; (3) practical support for low literacy and language barriers; (4) female facilitators/professionals especially for women from non-western backgrounds. Barriers to participation were similar to those reported in non-ethnic minority groups and included: (1) creating time in the workday to attend sessions; (2) inconsistent time intervals between sessions; (3) availability/location of rooms for sessions.

Although the overall quality of the literature was reported as moderate to high, the strength of evidence is severely limited by the small number of papers and participants, with the included ethnic minorities of limited relevance to the dominant ethnic groups in the UK workforce (South Asian and Black). Direct evidence relating to perceptions and attitudes of minority groups to wellbeing interventions in American workplaces is similarly lacking. US-based research suggests that interventions should focus on proactively addressing the issues of discrimination and inclusion to support employee health, as these are the issues that individuals report to have the greatest bearing on sense of wellbeing at work [21, 42].

#### 4.2.2 Grey literature

Although there was a wealth of grey literature on workplace health and wellbeing, only a small amount of literature referred to race or ethnicity. However, this literature did not go beyond reporting data by ethnicity [22] and providing recommendations for future research based on the lack of evidence on specific needs and intervention effectiveness in different ethnic groups [23, 24, 42]. There was no direct information on the attitudes and perceptions of ethnic minorities in relation to workplace health and wellbeing or reports on intervention effectiveness.

### 4.3 Supplementary narrative review

#### 4.3.1 Dimensions of ethnicity

When looking at designing interventions to better support ethnic minorities it is important to understand that ethnicity has many dimensions. Liu et al. [12] outlined five overlapping components: physical features, ancestry, language, culture, and religion. Furthermore, even in individuals who have the same ethnicity there may be different needs depending on gender, age generation, migration history and socio-economic background [25].

#### 4.3.2 Cultural adaptations of health and wellbeing interventions

There is a lack of clear evidence in the research literature on how best to adapt health promotion interventions to better support people from ethnic minorities. Yet adaptations have the potential to increase the effectiveness of interventions, by improving uptake and acceptability across the whole population [12].

Cultural Adaptation is “the systematic modification of an evidence-based treatment (or intervention protocol) to consider language, cultural, and context in such a way that it is compatible with the client’s cultural patterns, meaning, and values.” [26]. One of the earliest studies to look at cultural adaptation was based on the development of psychosocial treatments with a Hispanic population [27]. Bernal et al. [27] identified eight dimensions for treatment interventions that could be adapted; language, persons, metaphors, content, concepts, goals, methods, and context. More specifically related to health interventions, Netto et al. [28] outlined five principles for adapting health promotion interventions in the community. These principles have potential use within the workplace, with potential examples for this context outlined in Table 9.

**Table 9:** Five principles for adapting behavioural interventions with examples and potential crossover to the workplace (Adapted from Netto et al. [28])

Principle	Examples
1. Use of community resources to publicise the intervention and increase acceptability.	Use ethnic specific media and networks, community leaders and events to publicise events.
	Workplace adaptation: Utilise any current networks that are already in place for ethnic minorities in the workplace to publicise events or develop such networks.
2. Identify and address barriers to access and participation.	Tailor timing and location of events to BME women to account for caring responsibilities.
	Workplace adaptation: Arrange a discussion group to learn what barriers there are and how best they can be overcome.
3. Develop communication strategies that are sensitive to language use and information requirements.	Bilingual facilitators. Use spoken rather than written language to communicate with low literacy groups.
	Workplace adaptation: Work with people from ethnic minorities to adapt literature, using common and familiar terms. For example, a nutrition leaflet should include examples that use ethnic foods as well as western.
4. Work with cultural or religious values that either promote or hinder behavioural change.	Highlight compatibility of health promotion messages with religious beliefs.
	Workplace adaptation: As above.
5. Accommodate varying degrees of cultural identification.	Account for generation and migration history difference by more intensively exposing first-generation migrants to the intervention.
	Workplace adaptation: As above.

**Fig. 2:**
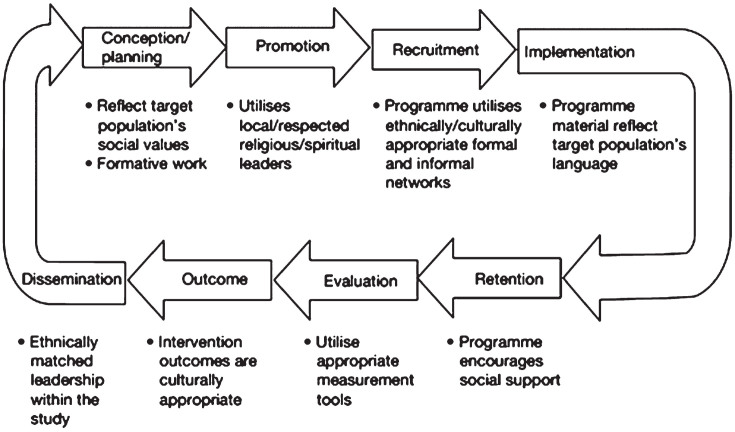
Programme theory of adapted health promotion interventions with examples of adaptations at each stage, reproduced from Liu et al. [12].

Liu et al. [12] used these five principles to set out a programme theory of adapted health promotion interventions (Fig. 2). These principles and theories were developed for use with community health interventions, yet few research studies have robustly tested the impact of implementing them in the community or workplace [12, 29]. In a recent review by Self et al. [30], 10 studies were identified as using culturally adapted motivational interviewing to promote behaviour change and reported that the culturally adapted intervention produced significantly better results for the primary outcome tested. However, data is still limited and has not been tested on health and wellbeing programmes in the workplace, yet they have the potential to provide a good starting point when designing or adapting health interventions for minority groups in the workplace.

#### 4.3.3 Community health, wellbeing and lifestyle interventions targeting UK ethnic minorities

Data on wellbeing and lifestyle interventions to improve health for ethnic minorities are scarce. There is slightly more research across the US, but in Europe this is limited. Nearly all studies in Europe are with South Asian groups and are community based [2]. The South Asian population is the fastest growing minority in Europe [31]. Learnings from qualitative literature that has looked at perceptions and attitudes including barriers and facilitators to leading a healthy lifestyle is scarce. A summary of common findings is outlined in Table 10.

**Table 10:** Common barriers and facilitators to leading a healthy lifestyle among ethnic groups in the UK

Health behaviour	Barriers and facilitators
General	Barriers
	•Financial constraints, childcare, time, accessing venues [34,35,36]
	•Language [2, 37]
	•Cultural and religious norms affect service utilisation [38]
	•Religious fatalistic attitudes [34,35] ‘whatever happens is because of God’s will’ [34]
	Facilitators
	•Gender specific facilities [39]
	•Type 2 Diabetes diagnosis [2]
	•Information available in mother tongue [39]
Physical Activity (PA)	Barriers
	•Practical challenges; Childcare, time, motivation [25, 40]
	•Suitable environment that is culturally appropriate for physical activity [34,36,40]
	•Lack of same sex venues and acceptability of western exercise clothing [2]
	•Cultural expectations and social responsibilities [40]
	•Prioritising work over PA to provide for the family [2]
	•Fear of racial harassment when exercising [2]
	•Religion and religious fatalism [40]
	Facilitators
	•Exercise class in safe environment i.e., place of worship [2]
	•Awareness of links between physical activity and health [40]
	•Previous interaction and engagement with health professionals [40]
Healthy eating	Barriers
	•Cultural barriers regarding serving and eating traditional foods [2, 41]
	•Acculturation - assimilation to the dominant culture [41]
	•Interpretation of national guidelines as “foreign and inapplicable” [41]
	•Taste over healthiness of food [41]
	•Un-achievability and undesirability of a healthy BMI [41]
	•Different perceptions over healthy body weight [2]L
	•Distrust of the health-care system [41]

#### 4.3.4 Useful research methods for programme design

When designing research with any group of individuals, one of the most crucial things is to talk to those who are going to use the service. This is often classed as ‘people-centred’ design or co-design and at the very least should include patient or public involvement. Patient and Public Involvement and Engagement (PPIE) entails research being carried out ‘with’ or ‘by’ members of the public, rather than ‘to’, ‘about’ or ‘for’ them [32]. Key stages of co-design include exploring the problems, identifying priorities, ideating, and finalising solutions tailored to the local context, implementing these solutions with and for the people for whom it is designed, ensuring the results meet their needs and are usable [33]. Research methods suitable for developing interventions using a co-design approach include, patient/public involvement (PPI), focus/discussion groups, interviews, surveys, questionnaires.

### 4.4 Strengths/limitations of review

To our knowledge this is the first scoping review to report the attitudes to and perceptions of ethnic minorities to workplace health and wellbeing interventions in the UK. The scoping review rigorously followed established review methodology [15], and included a quality appraisal of the included literature, ensuring a high standard. However, the supplementary review conducted to determine if there was relevant UK community-based literature was narrative, which lacks the systematic rigor of a scoping review, with no quality appraisal of the literature undertaken, and which may have missed relevant papers published outside of the UK.

Due to the very limited amount of literature in this area a broader research question may have been relevant to capture more learnings from other areas. Furthermore, the scoping review was restricted to content from the last 10 years to keep the data relevant to modern day practices, which may have missed potentially relevant studies conducted prior to 2012.

## 5 Conclusion

### 5.1 Key findings to inform future practice

Health and wellbeing and DE&I are deemed important in the UK workplace and can potentially support some of the key public health agendas in the UK around the health of the nation and health inequalities. Despite this there is very little research reporting the perceptions and attitudes of ethnic minorities, to determine how best to do this.

Current literature from both the workplace and community suggests a need for cultural adaptations to support recruitment, engagement and impact of health and wellbeing interventions. Some key adaptations that have potential to improve interventions for ethnic minorities include providing support with language barriers, availability of female deliverers, champions from similar ethnic backgrounds, a desire to be healthy, fears that weight gain might compromise family care, Type 2 Diabetes diagnosis, exercise classes in safe environment and an increased awareness of links between physical activity and health.

Many perceived barriers to accessing health and wellbeing support for ethnic minorities are similar to those experienced in the general population and include time and financial constraints. However, additional barriers such as, language, cultural and religious norms, lack of culturally suitable environments (to exercise), lack of same sex facilities/opportunities, fear of racial harassment, cultural traditions (food) and a distrust of western ways including health care and health guidelines.

A client-centred approach using methods such as co-design are key to enabling interventions to be designed and adapted in a way that is culturally sensitive and inclusive for the whole population.

### 5.2 Gaps in knowledge

The literature suggests a need for improvements in four key areas: (1) reporting of ethnic minorities in data relating to workplace health and wellbeing research, (2) more thorough review of perceptions and attitudes of ethnic minority workers in the UK, (3) design of culturally appropriate interventions that are tested for impact, and (4) testing of the effectiveness of culturally adapted interventions.

### 5.3 Summary

There is a clear lack of evidence relating to ethnic minorities and wellbeing in the workplace, particularly around perceptions and attitudes, with studies rarely reporting the ethnicity of participants or focusing on minority groups. This review was supplemented from literature (community) outside of the workplace, where there was some limited data. This has provided the researchers with a start point with some potentially useful insights around what might work and how this can be tested and built on in the future. Further research in this area is strongly recommended to build on the foundations of knowledge summarised in this paper.

## Ethical considerations

Not applicable.

## Informed consent

Not applicable.

## References

[1] Office of National Statistics. Ethnic group, England and Wales: Census 2021 [Internet]. 2022 [cited 22 September 2023]. Available from: Ethnic group, England and Wales - Office for National Statistics (ons.gov.uk)

[2] Patel N , Ferrer HB , Tyrer F , Wray P , Farooqi A , Davies MJ , et al. Barriers and Facilitators to Healthy Lifestyle Changes in Minority Ethnic Populations in the UK: a Narrative Review, J Racial and Ethnic Health Disparities. 2017;4(6):1107–1119. 10.1007/s40615-016-0316-y PMC5705764 27928772

[3] Muilwijk M , Nieuwdorp M , Snijder MB , Hof MHP , Stronks K , van Valkengoed IGM . The high risk for type 2 diabetes among ethnic minority populations is not explained by low-grade inflammation, SCI REP-UK. 2019;9(1):19871–8. 10.1038/s41598-019-56596-4 PMC6934847 31882814

[4] Dawkins NP , Yates T , Razieh C , Edwardson CL , Maylor B , Zaccardi F , Khunti K , Rowlands AV . Differences in Accelerometer-Measured Patterns of Physical Activity and Sleep/Rest Between Ethnic Groups and Age: An Analysis of UK Biobank, J Phys Act Health. 2022;19(1):37–46. 34826803 10.1123/jpah.2021-0334

[5] Lip GY , Barnett AH , Bradbury A , Cappuccio FP , Gill PS , Hughes E , Imray C , Jolly K , Patel K . Ethnicity and cardiovascular disease prevention in the United Kingdom: a practical approach to management, J Hum Hypertens. 2007;21(3):183–211. 17301805 10.1038/sj.jhh.1002126

[6] Ho FK , Gray SR , Welsh P , Gill JMR , Sattar N , Pell JP , Celis-Morales C . Ethnic differences in cardiovascular risk: examining differential exposure and susceptibility to risk factors, BMC Med. 2022;20(1):149. 35473626 10.1186/s12916-022-02337-w PMC9042646

[7] Baskin C , Zijlstra G , McGrath M , Lee C , Duncan FH , Oliver EJ , et al. Community-centred interventions for improving public mental health among adults from ethnic minority populations in the UK: a scoping review, BMJ Open. 2021;11(4):e041102.

[8] Mantovani N , Pizzolati M , Edge D . Exploring the relationship between stigma and help-seeking for mental illness in African-descended faith communities in the UK, Health Expect. 2017;20(3):373–384. 27124178 10.1111/hex.12464 PMC5433535

[9] Gov.uk. Ethnicity Facts & Figures [Internet]. 2022 [cited 22 September 2023]. Available from: https://www.ethnicity-facts-figures.service.gov.uk/workpay-and-benefits/employment/employment/latest.

[10] Black C Working for a healthier tomorrow. UK Dept. of Work and Pensions. 2008. Available from:https://www.gov.uk/government/publications/working-fora-healthier-tomorrow-work-and-health-in-britain.

[11] Dawson A , Phillips A Understanding ‘early exiters’, the case for a healthy ageing workforce strategy. The Physiological Society. 2022. Available from:https://demos.co.uk/wp-content/uploads/2022/11/Understanding-Early-Exiters-Demos.pdf.

[12] Liu J , Davidson E , Bhopal R , White M , Johnson M , Netto G , Deverill M , Sheikh A . Adapting health promotion interventions to meet the needs of ethnic minority groups: mixed-methods evidence synthesis, Health Technol Assess. 2012;16(44):1–469. 10.3310/hta16440 PMC4780992 23158845

[13] CIPD. Health and Wellbeing at Work. 2022. Available from:https://www.cipd.org/uk/knowledge/reports/healthwell-being-work/.

[14] Arksey H , O’Malley L . Scoping Studies: Towards a Methodological Framework, Int J Soc Res Methodol. 2005;8(1):19–32.

[15] Levac D , Colquhoun H , O’Brien KK . Scoping studies: advancing the methodology, Implement Sci. 2010;5:69. 20854677 10.1186/1748-5908-5-69 PMC2954944

[16] Critical Appraisal Skills Programme. Critical Appraisal Checklists [Internet]. 2023 [cited 22 September 2023]. Available from: https://casp-uk.net/casp-tools-checklists/.

[17] Richardson WS , Wilson MC , Nishikawa J , Hayward RS . The well-built clinical question: a key to evidence-based decisions, ACP J Club. 1995;123(3):A12–3. 10.7326/ACPJC-1995-123-3-A12 7582737

[18] Bertotti M , Dan-Ogosi I , Rao Mala . Workplace well-being in the London-Chinese business community, Int. J. Workplace Health Manag. 2017;10(2):86–100.

[19] Verburgh M , Verdonk P , Appelman Y , Brood-van Zanten M , Nieuwenhuijsen K . “I Get That Spirit in Me”-Mentally Empowering Workplace Health Promotion for Female Workers in Low-Paid Jobs during Menopause and Midlife, Int J Environ Res Public Health. 2020;17(18):6462. 32899848 10.3390/ijerph17186462 PMC7558098

[20] Verburgh M , Verdonk P , Appelman Y , Brood-van Zanten M , Hulshof C , Nieuwenhuijsen K . Workplace Health Promotion Among Ethnically Diverse Women in Midlife with a Low Socioeconomic Position, Health Educ Behav. 2022;49(6):1042–1055. 35125009 10.1177/10901981211071030 PMC9574907

[21] Connerley ML , Wu J , Combs GM , Milosevic I . Workplace Discrimination and the Wellbeing of Minority Women: Overview, Prospects, and Implications, Handbook on Well-Being of Working Women. 2016 17–31.

[22] Jackon C The evaluation of occupational health advice in Primary Care. The Health & Safety Executive. 2004. Available from:https://www.hse.gov.uk/research/rrpdf/rr242.pdf.

[23] National Institute of Clinical Excellence. Mental wellbeing at work (NG212) [Internet]. 2022 [cited 22 September 2023]. Available from: https://www.nice.org.uk/guidance/ng212.

[24] Newman R , Smith B , Wolpert M Putting science to work: Understanding what works for workplace mental health. Wellcome Trust. 2023. Available from:https://cms.wellcome.org/sites/default/files/2021-05/putting-science-work-understanding-workplacemental-health.pdf.

[25] Such E , Salway S , Copeland R , Haake S , Domone S , Mann S . A formative review of physical activity interventions for minority ethnic populations in England, J Public Health (Oxf). 2017;39(4):e265–e274. 27899479 10.1093/pubmed/fdw126 PMC6372170

[26] Castro FG , Barrera M , Holleran Steiker LK . Issues and Challenges in the Design of Culturally Adapted Evidence-Based Interventions, Annu Rev Clin Psychol. 2010;6(1):213–239. 20192800 10.1146/annurev-clinpsy-033109-132032 PMC4262835

[27] Bernal G , Bonilla J , Bellido C . Ecological validity and cultural sensitivity for outcome research: issues for the cultural adaptation and development of psychosocial treatments with Hispanics, J Abnorm Child Psychol. 1995;23(1):67–82. 7759675 10.1007/BF01447045

[28] Netto G , Bhopal R , Lederle N , Khatoon J , Jackson A . How can health promotion interventions be adapted for minority ethnic communities? Five principles for guiding the development of behavioural interventions, Health Promot Int. 2010;25(2):248–57. 20299500 10.1093/heapro/daq012

[29] Vincze L , Barnes K , Somerville M , Littlewood R , Atkins H , Rogany A , Williams LT . Cultural adaptation of health interventions including a nutrition component in Indigenous peoples: a systematic scoping review, Int J Equity Health. 2021;20(1):125. 34022886 10.1186/s12939-021-01462-x PMC8140502

[30] Self KJ , Borsari B , Ladd BO , Nicolas G , Gibson CJ , Jackson K , Manuel JK . Cultural adaptations of motivational interviewing: A systematic review, Psychol Serv. 2023;20(Suppl 1):7–18. 35130010 10.1037/ser0000619 PMC10161132

[31] Cainzos-Achirica M , Fedeli U , Sattar N , Agyemang C , Jenum AK , McEvoy JW , Murphy JD , Brotons C , Elosua R , Bilal U , Kanaya AM , Kandula NR , Martinez-Amezcua P , Comin-Colet J , Pinto X . Epidemiology, risk factors, and opportunities for prevention of cardiovascular disease in individuals of South Asian ethnicity living in Europe, Atherosclerosis. 2019;286:105–113. 31128454 10.1016/j.atherosclerosis.2019.05.014 PMC8299475

[32] National Institute for Health Research. Briefing notes for researchers - public involvement in NHS, health and social care research [Internet]. 2021 [cited 22 September 2023]. Available from: https://www.nihr.ac.uk/documents/briefing-notes-forresearchers-public-involvement-in-nhs-health-and-socialcare-research/27371.

[33] Agency for Clinical Innovation. A guide to build co–design capability. New South Wales Government. 2019. Available from:https://aci.health.nsw.gov.au/_data/assets/pdf_file//40/Guide-Build-Codesign-Capability.pdf.

[34] Greenhalgh T , Helman C , Chowdhury AM . Health beliefs and folk models of diabetes in British Bangladeshis: a qualitative study, BMJ. 1998;316(7136)978–83. 9550958 10.1136/bmj.316.7136.978 PMC28502

[35] Grace C , Begum R , Subhani S , Kopelman P , Greenhalgh T . Prevention of type 2 diabetes in British Bangladeshis: qualitative study of community, religious, and professional perspectives, BMJ. 2008;337:a1931. 18984633 10.1136/bmj.a1931 PMC2659954

[36] Choudhury SM , Brophy S , Williams R . Understanding and beliefs of diabetes in the UK Bangladeshi population, Diabetic Med. 2009;26:636–40. 19538240 10.1111/j.1464-5491.2009.02741.x

[37] Gumber L . Knowledge gaps and other barriers in type 2 diabetes management: Findings from interviews with South Asian women, Diabetes & Primary Care. 2014;16:86–91.

[38] Alam R , Speed S , Beaver K . A scoping review on the experiences and preferences in accessing diabetes-related healthcare information and services by British Bangladeshis, Health Soc Care Comm. 2012;20:155–71. 10.1111/j.1365-2524.2011.01027.x 21883609

[39] Sohal T , Sohal P , King-Shier KM , Khan NA . Barriers and Facilitators for Type-2 Diabetes Management in South Asians: A Systematic Review, PLoS One. 2015;10(9):e0136202. 26383535 10.1371/journal.pone.0136202 PMC4575130

[40] Ige-Elegbede J , Pilkington P , Gray S , Powell J . Barriers and facilitators of physical activity among adults and older adults from Black and Minority Ethnic groups in the UK: A systematic review of qualitative studies, Prev Med Rep. 2019;15:100952. 31367514 10.1016/j.pmedr.2019.100952 PMC6656684

[41] Majumdar A , Sarbah-Yalley SEA . Facilitators and barriers to making healthy lifestyle choices: a qualitative exploration in a UK-based Ghanaian population, Lancet. 2019;S71(394).

[42] Szczepura A , Gumber G , Clay D , Davies R , Elias P , Johnson M , Walker I , Owen D Review of the occupational health and safety of Britain’s ethnic minorities. Health & Safety Executive. 2004. Available from: http://www.hse.gov.uk/research/rrpdf/rr221.pdf.

